# Supplementary material for: TANGO1 inhibitors reduce collagen secretion and limit tissue scarring
Source: Nat Commun. 2024 Apr 24;15:3302. doi: 10.1038/s41467-024-47004-1 (PMC11043333; doi:10.1038/s41467-024-47004-1)
Supplement: Supplementary file 1 — Supplementary Information [file 41467_2024_47004_MOESM1_ESM.pdf]

Supplementary Figures

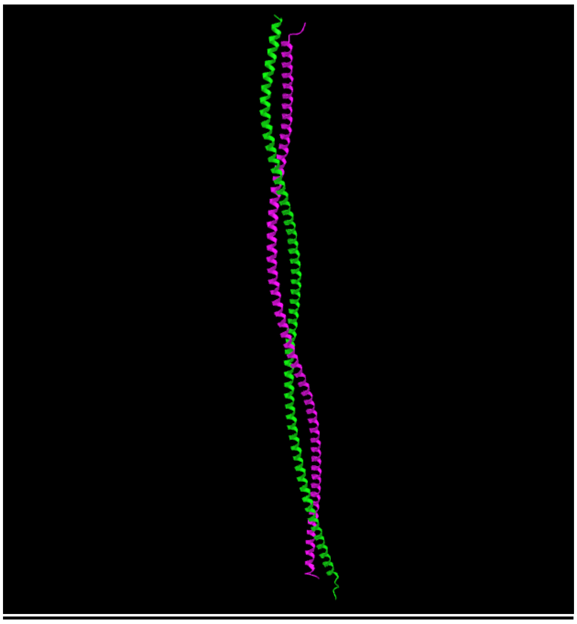

Supplementary figure 1

Structure prediction of the TANGO1-cTAGE5 dimeric region from AlphaFold2.

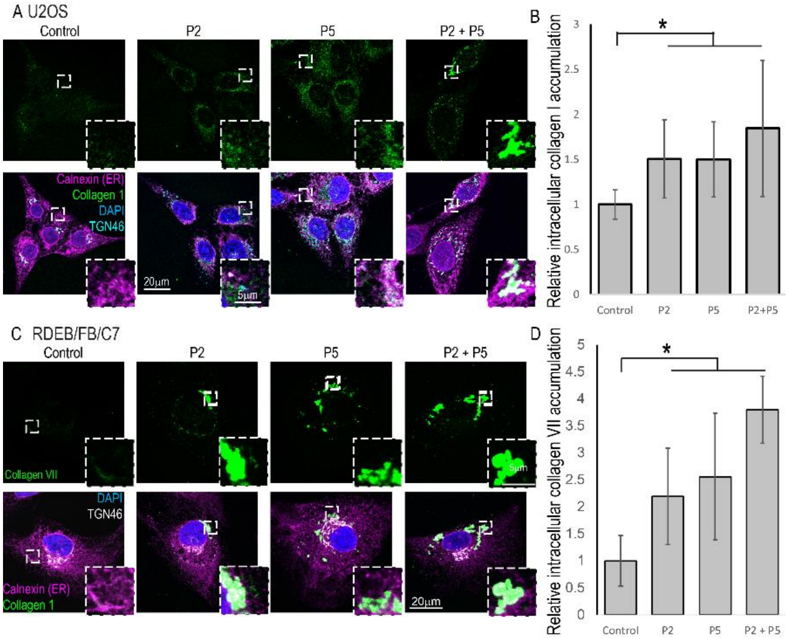

Supplementary figure 2. Peptide inhibitors bring about collagen retention in the ER.

(**A**) U2OS cells treated with peptides show accumulations of collagen I (green) in the ER (magenta). (**B**) Quantification of the intracellular collagen I accumulation (normalized to control) (**C**) RDEB/FB/C7 cells treated with peptides show accumulations of collagen VII (green) in the ER (magenta). (**D**) Quantification of the intracellular collagen VII accumulation (normalized to control). Insets show a magnified section of collagen accumulations. Scale bars 20 μm or insets 5 μm. *$p<0.05$, Students' t-test.

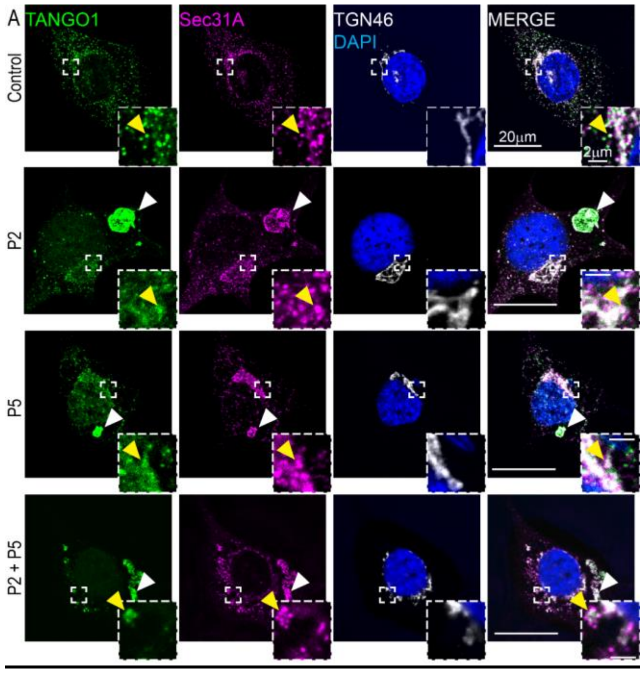

<u>Supplementary figure 3</u>. Peptide inhibitors do not affect TANGO1 localization to ERES or collagen recruitment.

(**A**) RDEB/FB/C7 cells treated with peptides show TANGO1 (green) recruitment to accumulations of collagen (white arrowheads), as reported before. TANGO1 puncta are colocalised with ERES markers Sec31A (magenta). Insets show a magnified section of collagen accumulations, yellow arrowheads highlight TANGO1 and Sec31A puncta. Scale bars 20 µm or insets 2 µm.

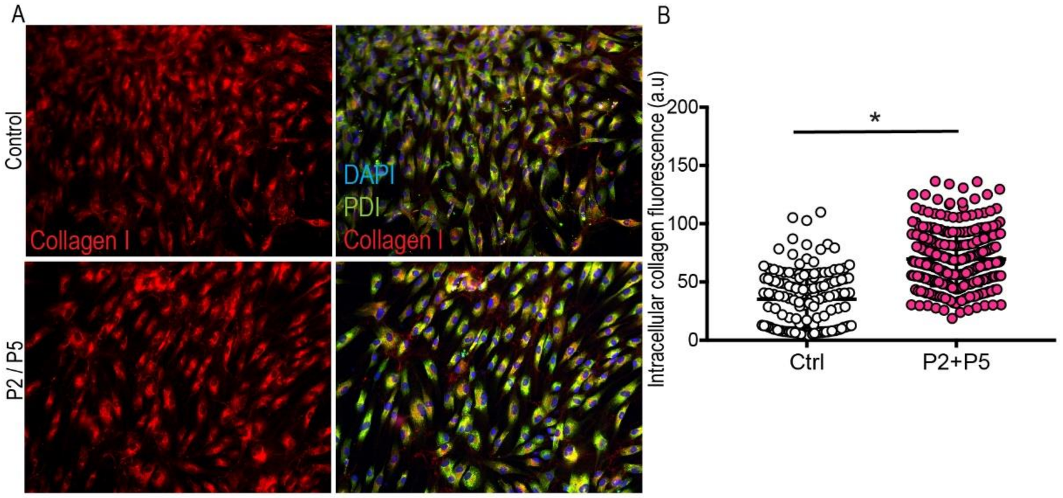

<u>Supplementary figure 4</u>. Peptide inhibitors cause intracellular accumulation of collagen

(**A**) Primary skin fibroblasts after 20 h of incubation without (Ctrl) or with the combination of P2+P5 at 40 µM and then stained to visualise collagen I (red), PDI (green) Scale bar: 50 µm. (**B**) Quantification of intensity of collagen I staining in control (black) vs treated (magenta) fibroblasts in two independent experiments each, from two individuals showed a significant but mild difference (* $p < 0.0001$, Mann Whitney U, two-tailed). N = 141 (Ctrl) / 329 (P2 + P5) cells. Data shown as mean ± SD.

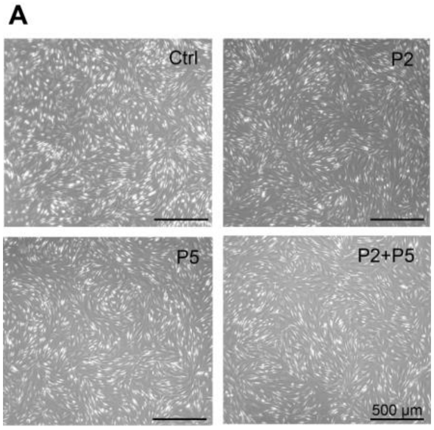

Supplementary Figure 5. Peptides do not induce changes in morphology of primary human skin fibroblasts

(A) Representative phase contrast images of primary skin fibroblasts after 20 h of incubation without (Ctrl) and with either P2, P5 or the combination of P2+P5 at 40 µM without serum. Bar: 500 µm.

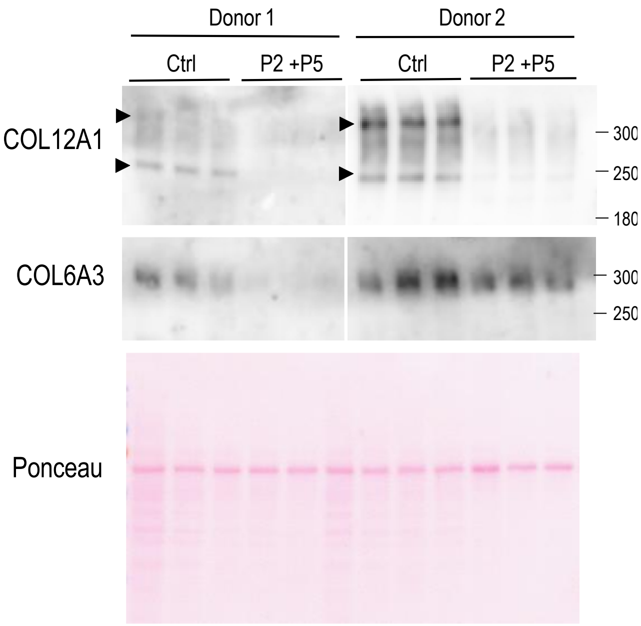

Supplementary Figure 6. Peptides reduce secretion of COL6A1 and COL12A1.

Western blots of supernatants from fibroblasts incubated for 20 h in the absence of serum without (Ctrl) or with 40 µM each of P2+P5. Peptide treatment strongly reduced amounts of secreted COL6A1 and of COL12A1. The long and short splice variants of COL12A1 are indicated by black arrow heads. Exposure times of blots for the two donors differed. Ponceau staining of the corresponding cell lysates indicates comparable cell numbers. N = 2 donors.

Gene names	Protein IDs	Protein names	$-Log_{10}$ T test P-value (P2P5 vs. Control)	Log_2 FC (P2P5 vs. Control)
VCAN	P13611	Versican core protein	1.81170	-5.19527
FBN1	P35555	Fibrillin-1	4.01801	-4.67462
COL12A1	Q99715	Collagen alpha-1(XII) chain	4.37419	-4.67456
COL3A1	P02461	Collagen alpha-1(III) chain	3.92008	-3.59692
COL5A1	P20908	Collagen alpha-1(V) chain	3.42365	-3.12562
COL6A3	P12111	Collagen alpha-3(VI) chain	3.13966	-3.01714
CTSB	P07858	Cathepsin B	2.05424	-2.54762
COL6A2	P12110	Collagen alpha-2(VI) chain	1.67596	-2.31061
LAMA4	Q16363	Laminin subunit alpha-4	2.61542	-2.25661
CLU	P10909	Clusterin	1.86799	-2.23954
PDIA3	P30101	Protein disulfide-isomerase A3	1.95555	-2.19724
C1S	P09871	Complement C1s subcomponent	1.90802	-1.85249
FSTL1	Q12841	Follistatin-related protein 1	3.09804	-1.83823
PSAP	P07602	Prosaposin	2.03952	-1.74644
TMSB4X	P62328	Thymosin beta-4	1.80659	-1.60465
COL1A1	P02452	Collagen alpha-1(I) chain	3.47620	-1.55965
YWHAZ	P63104	14-3-3 protein zeta/delta	2.41210	-1.47430
RCN1	Q15293	Reticulocalbin-1	1.71327	-1.36175
COL1A2	P08123	Collagen alpha-2(I) chain	4.02213	-1.33409
MIA3	Q5JRA6	Melanoma inhibitory activity protein 3	1.66826	8.49901
ACTB	P60709	Actin	2.92456	1.91751
ENO1	P06733	Alpha-enolase	2.38767	1.85727
CFL1	P23528	Cofilin-1	2.54660	1.63683
LGALS1	P09382	Galectin-1	3.19137	1.63093
PGK1	P00558	Phosphoglycerate kinase 1	1.69194	1.40227
SERPINE1	P05121	Plasminogen activator inhibitor 1	3.14065	1.33331
VIM	P08670	Vimentin	2.92202	1.31643

Hyposecreted in P2 + P5 treated   Hypersecreted in P2 + P5 treated

Supplementary Table 1. List of selected proteins hypo- (in blue) or hypersecreted (in red) in supernatants of primary human skin fibroblasts treated with P2+P5 versus control (secretome).

Gene names	Protein IDs	Protein names	$-Log_{10}$ T test P-value (P2P5 vs. Control)	Log_2 FC (P2P5 vs. Control)
MMP1	P03956	Interstitial collagenase	1.70477	0.83577
SERPINE2	P07093	Glia-derived nexin	1.24836	0.71191
TIMP1	P01033	Metalloproteinase inhibitor 1	1.07280	0.59529
SPARC	P09486	SPARC	1.24367	0.48131
ANXA2	P07355	Annexin A2	0.59280	0.41588
TGFBI	Q15582	Transforming growth factor-beta-induced protein ig-h3	1.21532	0.41117
CYR61	O00622	Protein CYR61	0.73704	0.29120
FBLN2	P98095	Fibulin-2	0.70771	0.27581
CST3	P01034	Cystatin-C	2.50978	0.26645
TIMP2	P16035	Metalloproteinase inhibitor 2	0.14051	0.18951
MMP2	P08253	72 kDa type IV collagenase	0.21836	0.16330
PKM	P14618	Pyruvate kinase PKM	0.05721	0.04434
CCDC80	Q76M96	Coiled-coil domain-containing protein 80	0.00974	0.00646
COL6A1	P12109	Collagen alpha-1(VI) chain	0.07256	-0.05182
LUM	P51884	Lumican	0.89805	-0.21413
POSTN	Q15063	Periostin	0.35686	-0.38590
SOD1	P00441	Superoxide dismutase [Cu-Zn]	0.00000	-0.57423
ECM1	Q16610	Extracellular matrix protein 1	1.65417	-0.88092

Supplementary Table 2. List of selected proteins from the secretome of primary human skin fibroblasts that show no significant changes between control conditions and those treated with P2+P5.
