# Supplementary figures and images for: TANGO1 inhibitors reduce collagen secretion and limit tissue scarring
Source: Nat Commun. 2024 Apr 24;15:3302. doi: 10.1038/s41467-024-47004-1 (PMC11043333; doi:10.1038/s41467-024-47004-1)

Figure 1F

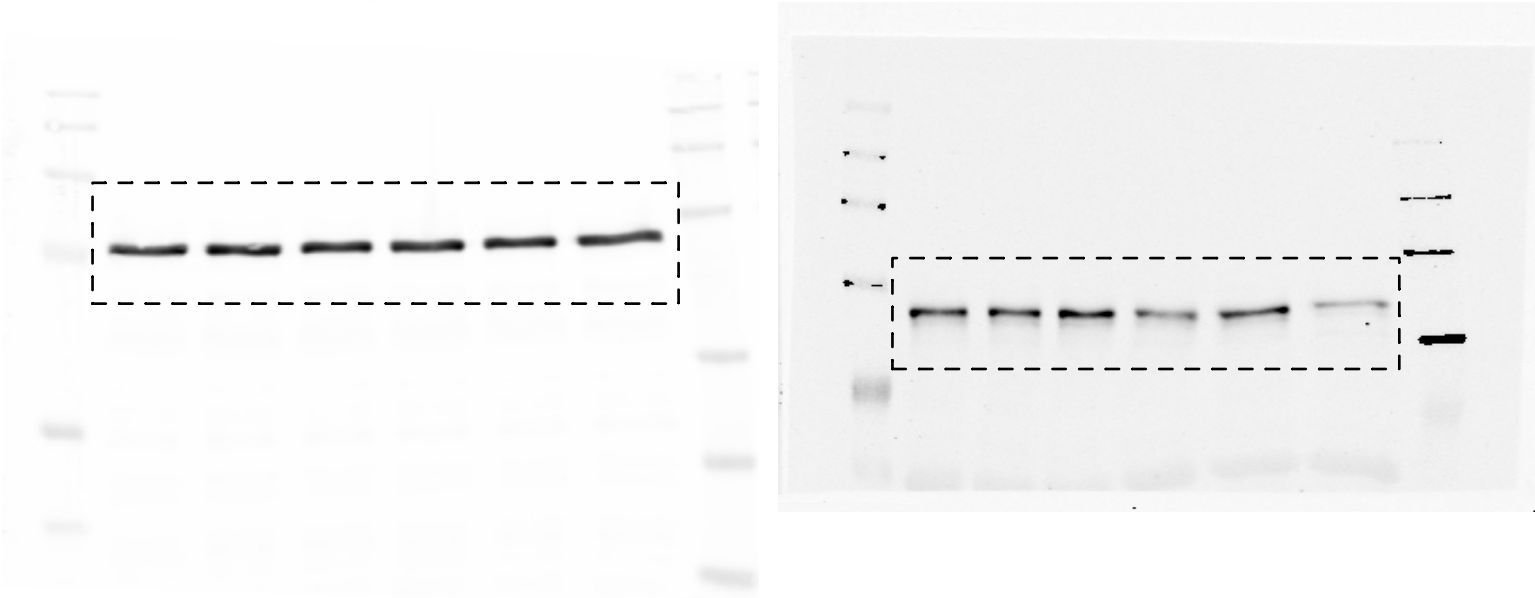

Figure 1G

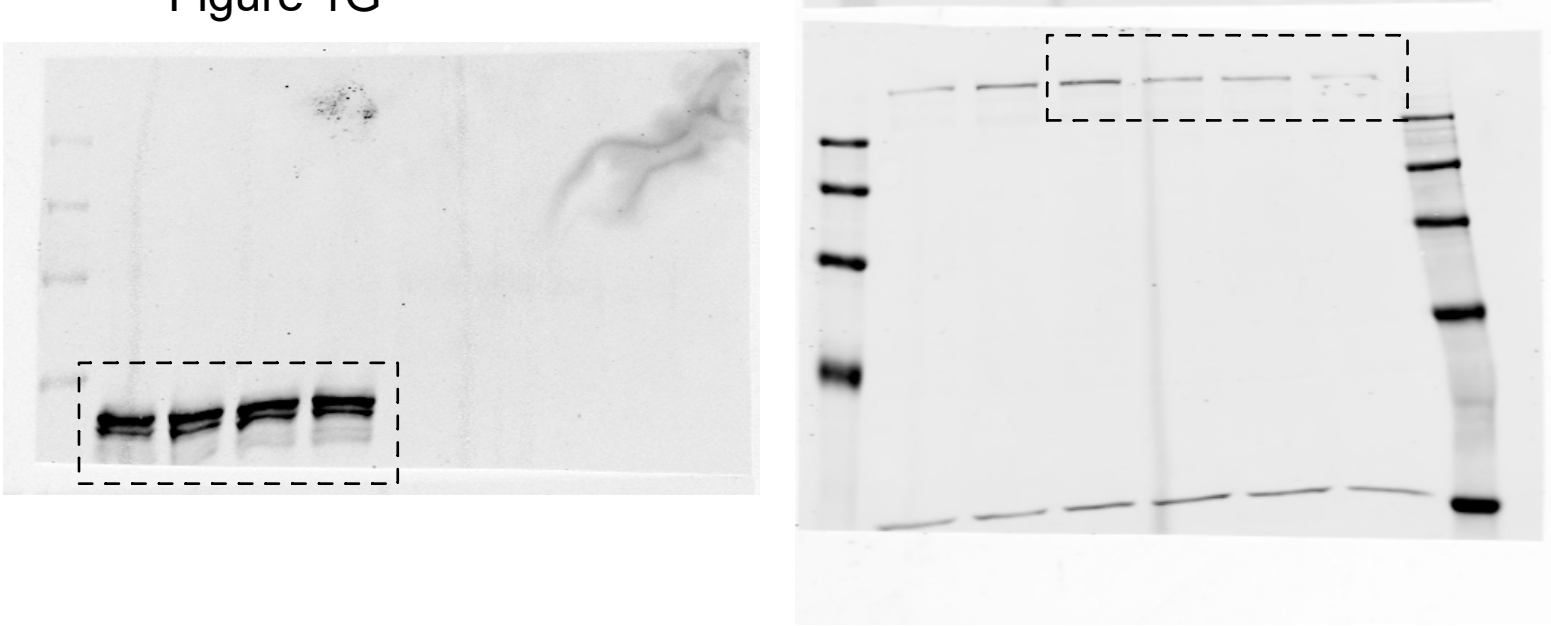

Supplement: Supplementary file 4 — Source Data [file 41467_2024_47004_MOESM4_ESM.zip › SOURCE DATA/444095_3_rel_ms_0_s9vtrc.pdf]
